# Supplementary material for: Inhibitory properties of crude microalgal extracts on the in vitro replication of cyprinid herpesvirus 3
Source: Sci Rep. 2021 Nov 30;11:23134. doi: 10.1038/s41598-021-02542-2 (PMC8633293; doi:10.1038/s41598-021-02542-2)
Supplement: Supplementary file 1 — Supplementary Information. [file 41598_2021_2542_MOESM1_ESM.pdf]

Table S1: Overview of the microorganisms used for antiviral screening with information about the source and cultivation medium.

Species	Division	Source, strain number	Cultivation conditions (medium, T, CO_2, PFD)
A. platensis	Cyanobacteria	NIES-39	modified *A. platensis* medium [74], 35 °C, 3 % CO_2, 180 µmol m^{-2} s^{-1}
N. punctiforme	Cyanobacteria	SAG 69.79	BG11, 25 °C, 3 % CO_2, 40 µmol m^{-2} s^{-1}
C. reinhardtii	Chlorophyta	CC-125	TAP, 22 °C, 2.6 % CO_2, 100 µmol m^{-2} s^{-1}
C. kessleri	Chlorophyta	SAG 211-11g	BG11, 25 °C, 3 % CO_2, 40 µmol m^{-2} s^{-1}
H. pluvialis	Chlorophyta	SAG 34-1a	M1b, 25 °C, 3 % CO_2, 40 µmol m^{-2} s^{-1}
S. obliquus	Chlorophyta	SAG 276-3d	M1b, 25 °C, 3 % CO_2, 40 µmol m^{-2} s^{-1}

CC: Chlamydomonas Resource Center, University of Minnesota
NIES: Microbial Culture Collection at the National Institute for Environmental Studies, Japan
SAG: Culture Collection of Algae at Göttingen University
PFD: photon flux density

Table S2: Thermal cycling protocol used for real-time PCR for the quantitative detection of CyHV-3 copy numbers.

Step		Temperature	Duration
1	Polymerase Activation and DNA Denaturation	95 °C	5 min
2	Denaturation	95 °C	5 sec
3	Annealing / Extension and Plate Read	60 °C	30 sec
	40 Cycles (Repetition of step 2 and 3)		

Table S3: Overview of the primers and probes used for the quantitative detection of viral and cellular DNA using qPCR according to Gilad *et al.* [76].

	Primer / Probe	Sequence (5‘ → 3‘)	Size, bp
CyHV-3	CyHV-3 86 f	GACGCCGGAGACCTTGTG	78
	CyHV-3 163 r	CGGGTTCTTATTTTTGTCCTTGTT	
	CyHV-3 109 p	CTTCCTCTGCTCGGCGAGCACG	
Glucokinase	CgGluc 162 f	ACTGCGAGTGGAGACACATGAT	69
	CgGluc 230 r	TCAGGTGTGGAGCGGACAT	
	CgGluc 185 p	AAGCCAGTGTCAAAATGCTGCCCACT	

Dose-response curves for the basic and enhanced experiments

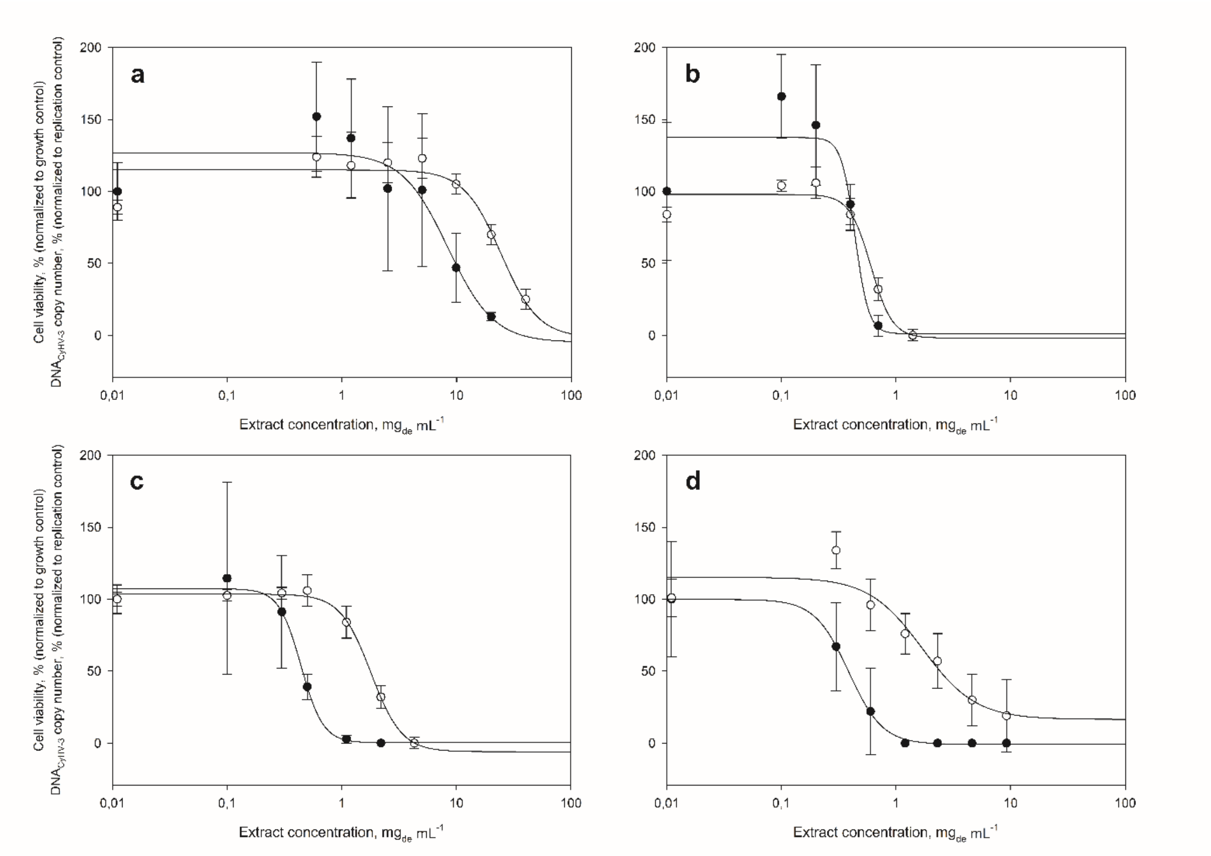

Figure S1: Cytotoxic and virus inhibiting effects of basic *C. kessleri* (a), *S. obliquus* (b), *N. punctiforme* (c) and *C. reinhardtii* (d) extracts, shown as normalized cell viability (white circles, based on growth control) and normalized CyHV-3 copy number (black circles, based on replication control) depending on the extract concentration. The values shown are the mean values of six biological replicates ± SD. Dose-response curves were approximated by regression using the *Four Parameter Logistic Curve* (Sigma Plot).

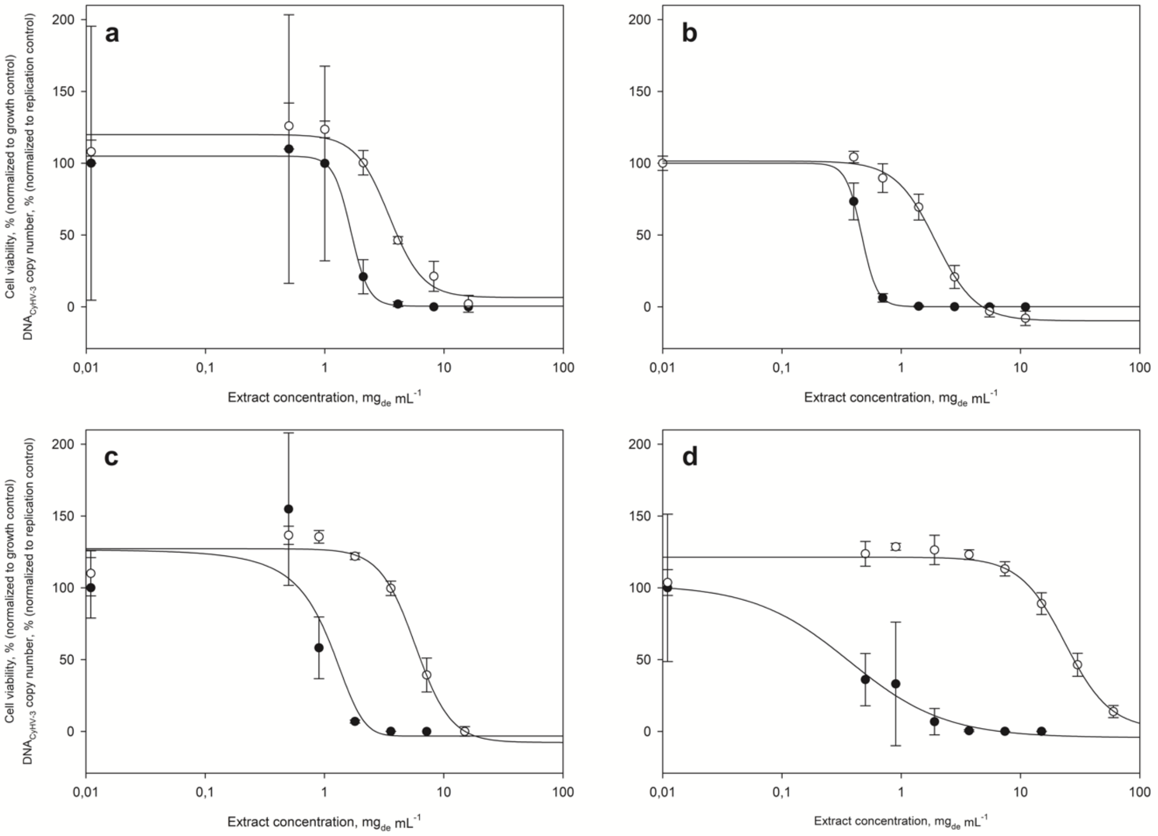

Figure S2: Cytotoxic and virus inhibiting effects of enhanced *A. platensis* (a), *N. punctiforme* (b), *C. reinhardtii* (c) and *H. pluvialis* (d) extracts, shown as normalized cell viability (white circles, based on growth control) and normalized CyHV-3 copy number (black circles, based on replication control) depending on the extract concentration. The values shown are the mean values of six biological replicates ± SD. Dose-response curves were approximated by regression using the *Four Parameter Logistic Curve* (Sigma Plot).

Interestingly, especially in samples containing low extract concentrations, normalized cell viabilities of above 100 % were determined using the MTT assay, suggesting viabilities better than observed in replication controls. This phenomenon has already been found when investigating the influence of cell-toxic substances by the MTT assay which is based on the metabolic activity of the cells [59, 60]. It could be explained by the hypothesis of hormesis, constituting a positive biological response to low concentrations of toxic substances [61]. Hormesis is part of many dose-response relationships and can lead to a maximal stimulation of 30 to 60 % compared to control values [62]. Therefore, it is possible that the same extracts showing a cytotoxic effect at high concentrations, had a slightly stimulating effect on the metabolism of the CCB cells at exceptionally low concentrations, resulting in increased viability. Another possibility is the dilution of the extracts and thus the partly different concentration of cell damaging DMSO in the samples. Both, the replication control and the undiluted extract contained 1% DMSO from the redissolution process. Since the concentration of DMSO decreased with the consecutive extract dilutions, the latter could have less cell-damaging effects at low extract concentrations compared to the replication control. Despite this and the relatively high standard deviation of this biological assay, the MTT assay enabled the evaluation of cytotoxic effects of the extracts on the host cells. After the 72 h incubation periods, the cell viability of the replication control was mostly comparable to that of the growth control. Therefore, it can be assumed that the affected vitalities of the samples were actually consequences of the addition of extract.

Table S4: Overview of the total protein content of the extracts. Protein quantification was carried out using the Bradford assay according to the manufacturer specifications. A dilution series of bovine serum albumin (BSA) was used as standard. The means of three replicates ± SD are given.

Species, basic extraction	Total protein content, µg mg_{de}^{-1}	Species, enhanced extraction*	Total protein content, µg mg_{BTM}^{-1}
Arthrospira platensis	99 ± 22	*Arthrospira platensis**	93 ± 12
Haematococcus pluvialis	57 ± 2.0	*Haematococcus pluvialis**	76 ± 4.9
Chlamydomonas reinhardtii	57 ± 1.1	*Chlamydomonas reinhardtii**	52 ± 4.1
Scenedesmus obliquus	52 ± 3.0		
Nostoc punctiforme	51 ± 8.1		
Chlorella kessleri	46 ± 2.6		

* Enhanced extraction conditions (100 °C, EtOH : H_2O, 80 : 20 v/v)

Table S5: Overview of the total lipid content of the extracts. The quantification was carried out according to the method described by Beck *et al.* for the extraction of fatty acids and the subsequent gravimetric analysis of the dried residues [75].

Species, basic extraction	Total lipid content, µg mg_{de}^{-1}	Species, enhanced extraction*	Total lipid content, µg mg_{de}^{-1}
Chlamydomonas reinhardtii	287	*Chlamydomonas reinhardtii**	267
Arthrospira platensis	113	*Arthrospira platensis**	94
Haematococcus pluvialis	100	*Haematococcus pluvialis**	149
Chlorella kessleri	69		
Nostoc punctiforme	65		
Scenedesmus obliquus	33		

* Enhanced extraction conditions (100 °C, EtOH : H_2O, 80 : 20 v/v)
